# Supplementary material for: Representations and decodability of diverse cognitive functions are preserved across the human cortex, cerebellum, and subcortex
Source: Commun Biol. 2022 Nov 14;5:1245. doi: 10.1038/s42003-022-04221-y (PMC9663596; doi:10.1038/s42003-022-04221-y)
Supplement: Supplementary file 2 — Supplementary Information [file 42003_2022_4221_MOESM2_ESM.pdf]

Representations and decodability of diverse cognitive functions are preserved across the human cortex, cerebellum, and subcortex

Tomoya Nakai, Shinji Nishimoto

Supplementary Information

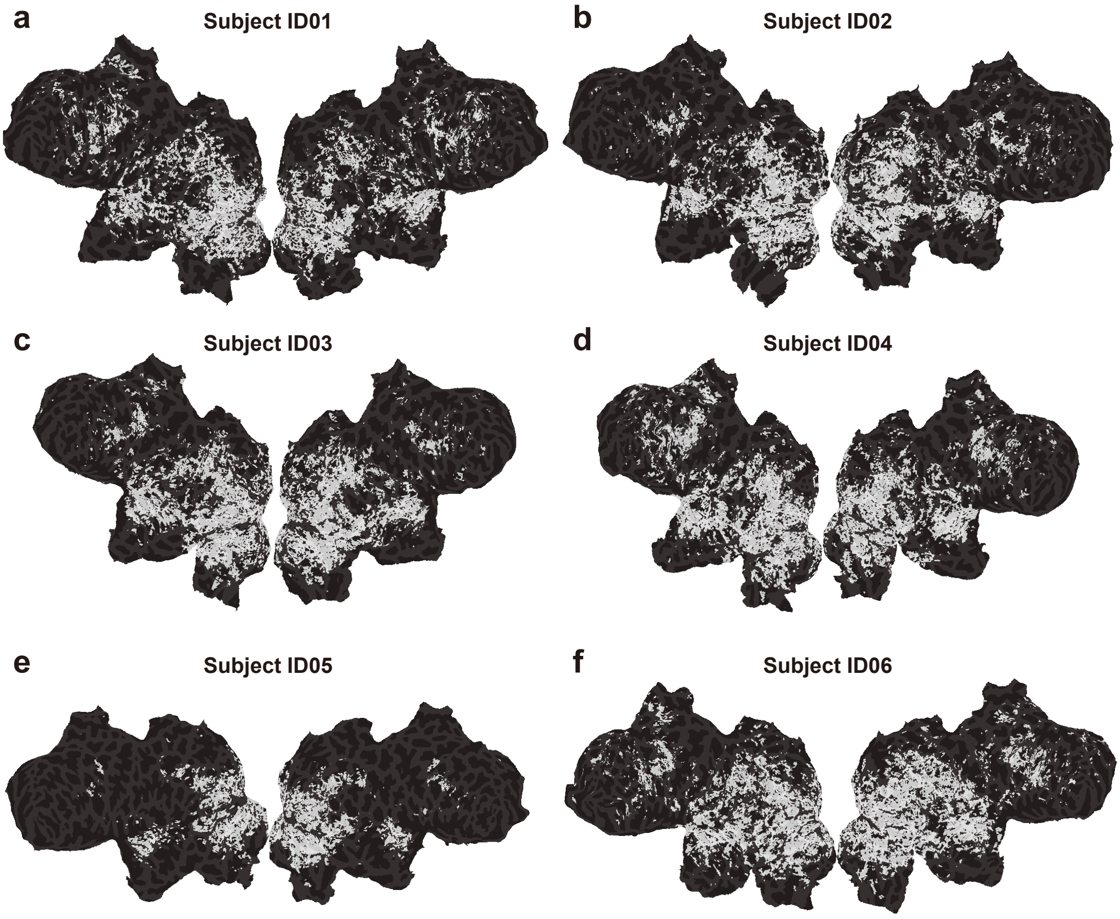

Supplementary Figure 1. Cortical map of sensorimotor voxels predicted by the regressor features (threshold, $r = 0.3$), mapped on the flattened cortical sheets of subjects ID01–ID06.

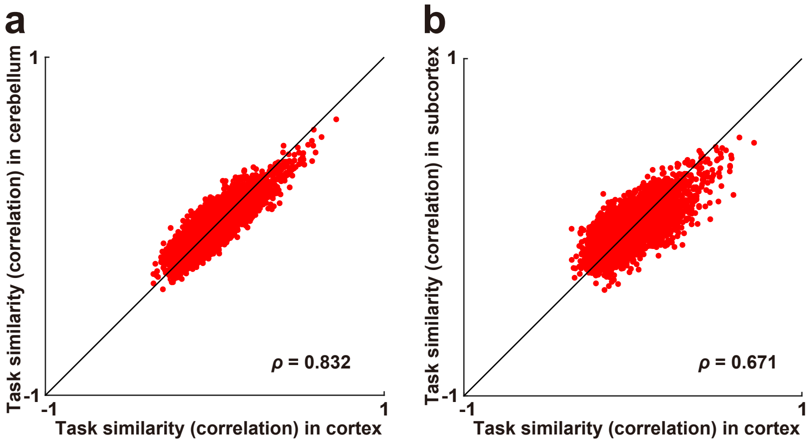

Supplementary Figure 2. Scatter plot of task similarities **a** for the cortex and cerebellum, and **b** for the cortex and subcortex after excluding sensorimotor voxels.

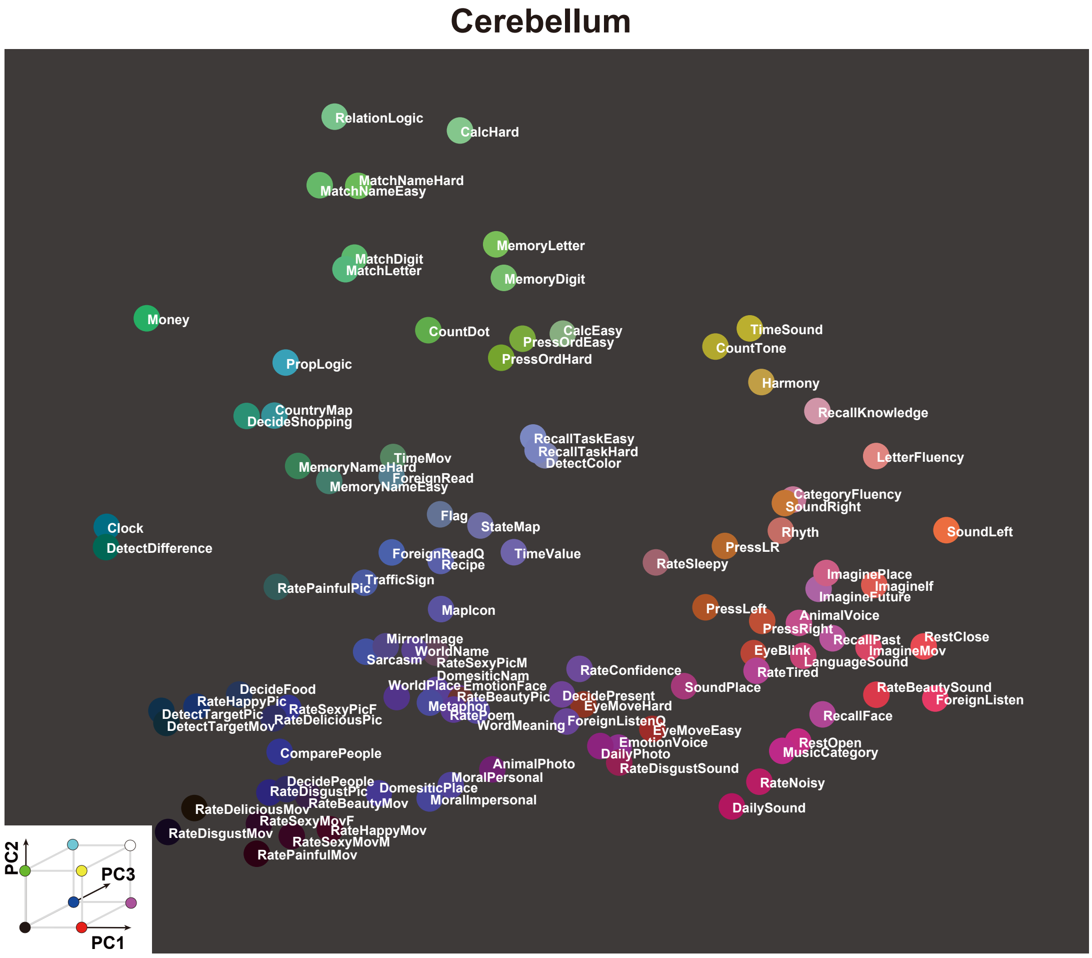

Supplementary Figure 3. Visualization of task structures in the cerebellum. All tasks are labeled.

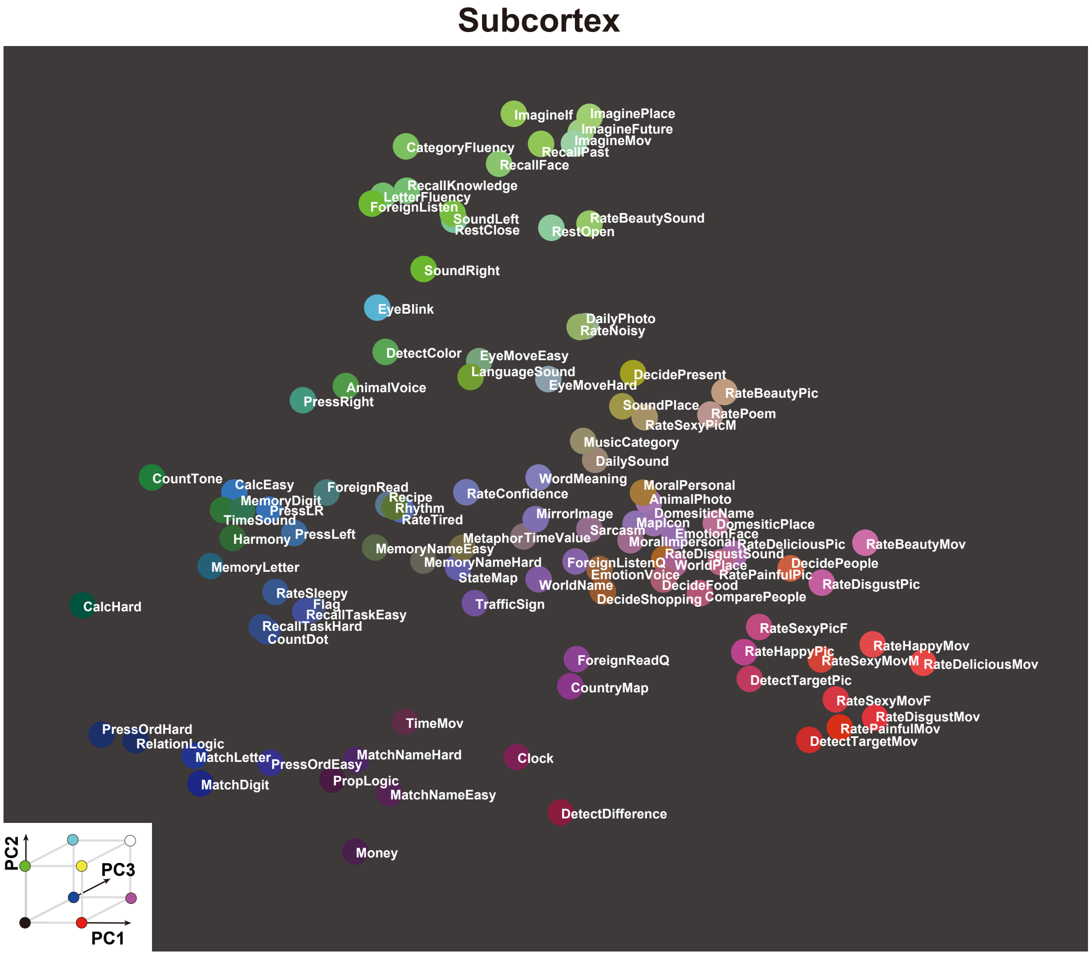

Supplementary Figure 4. Visualization of task structures in the subcortex. All tasks are labeled.

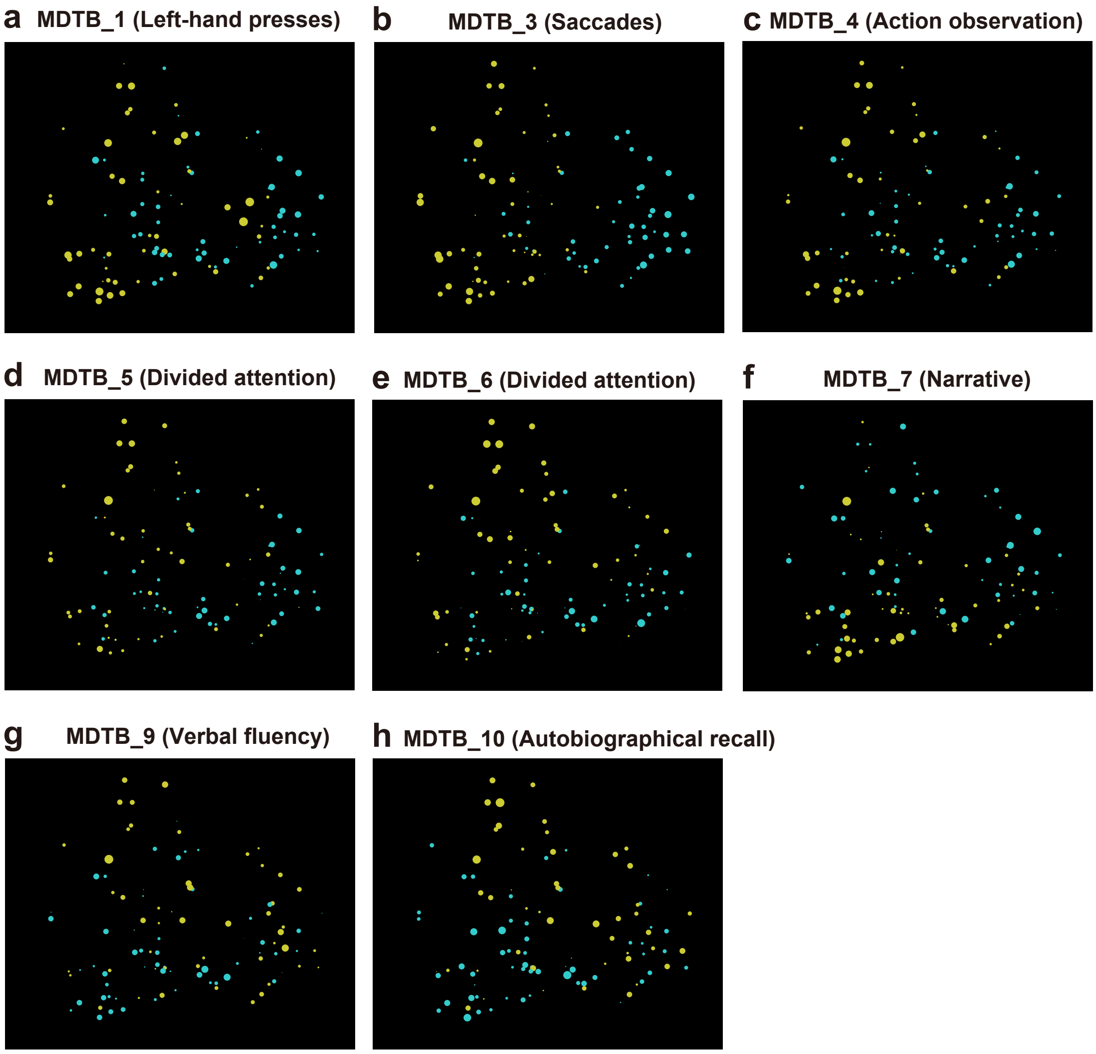

Supplementary Figure 5. Task selectivity for voxels in the functional subregions in the cerebellum (multi-domain task battery (MDTB) parcellation in the cerebellum[1], mapped onto the same two-dimensional cognitive space as (Fig. 3a), for subject ID01. Tasks with positive and negative weight values were denoted in yellow and cyan, respectively. The circle size was modulated based on the absolute weight value.

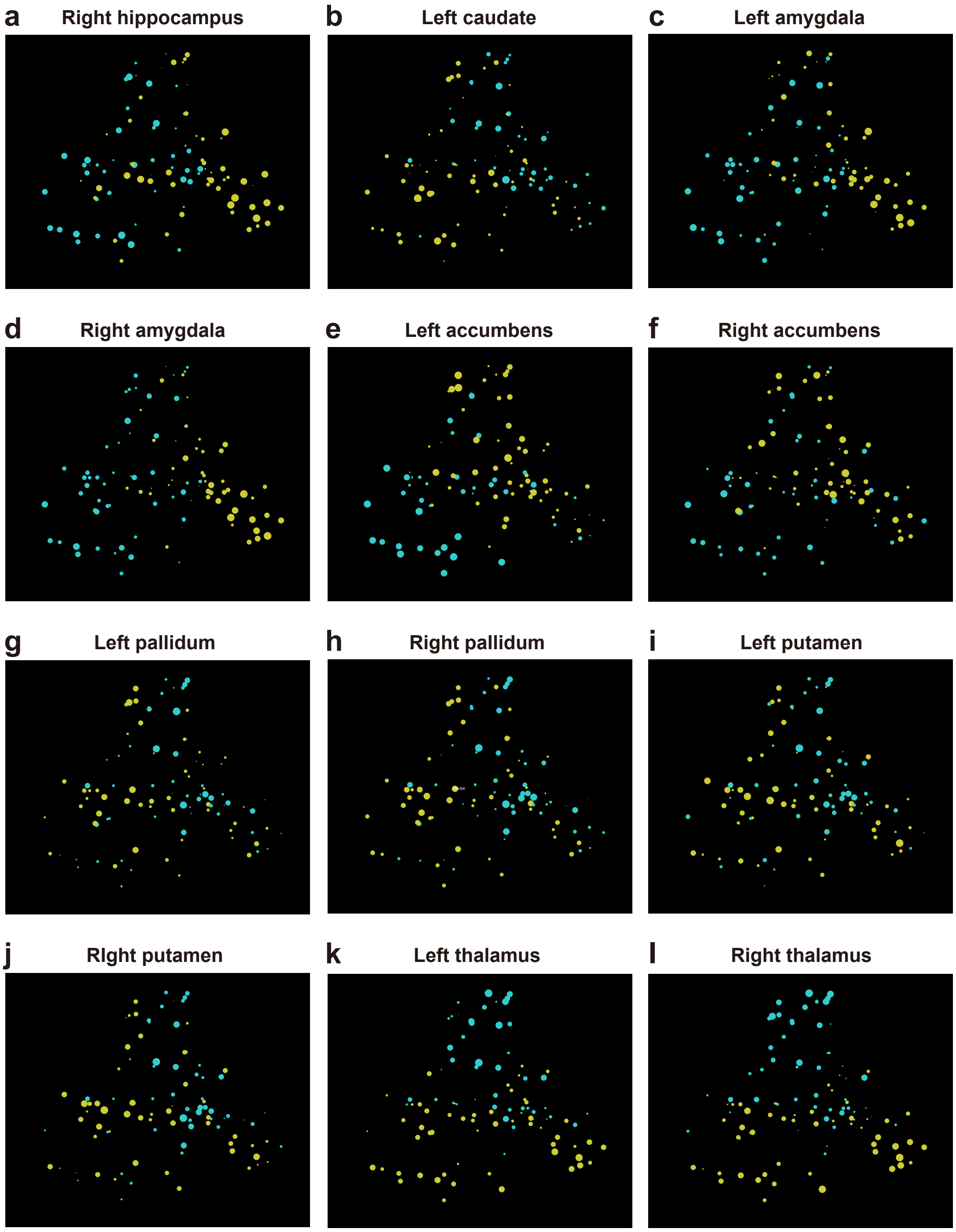

Supplementary Figure 6. Task selectivity for voxels in the anatomical regions of interests in the subcortex, mapped onto the same two-dimensional cognitive space as (Fig. 4a), for subject ID01. Tasks with positive and negative weight values were denoted in yellow and cyan, respectively. The circle size was modulated based on the absolute weight value.

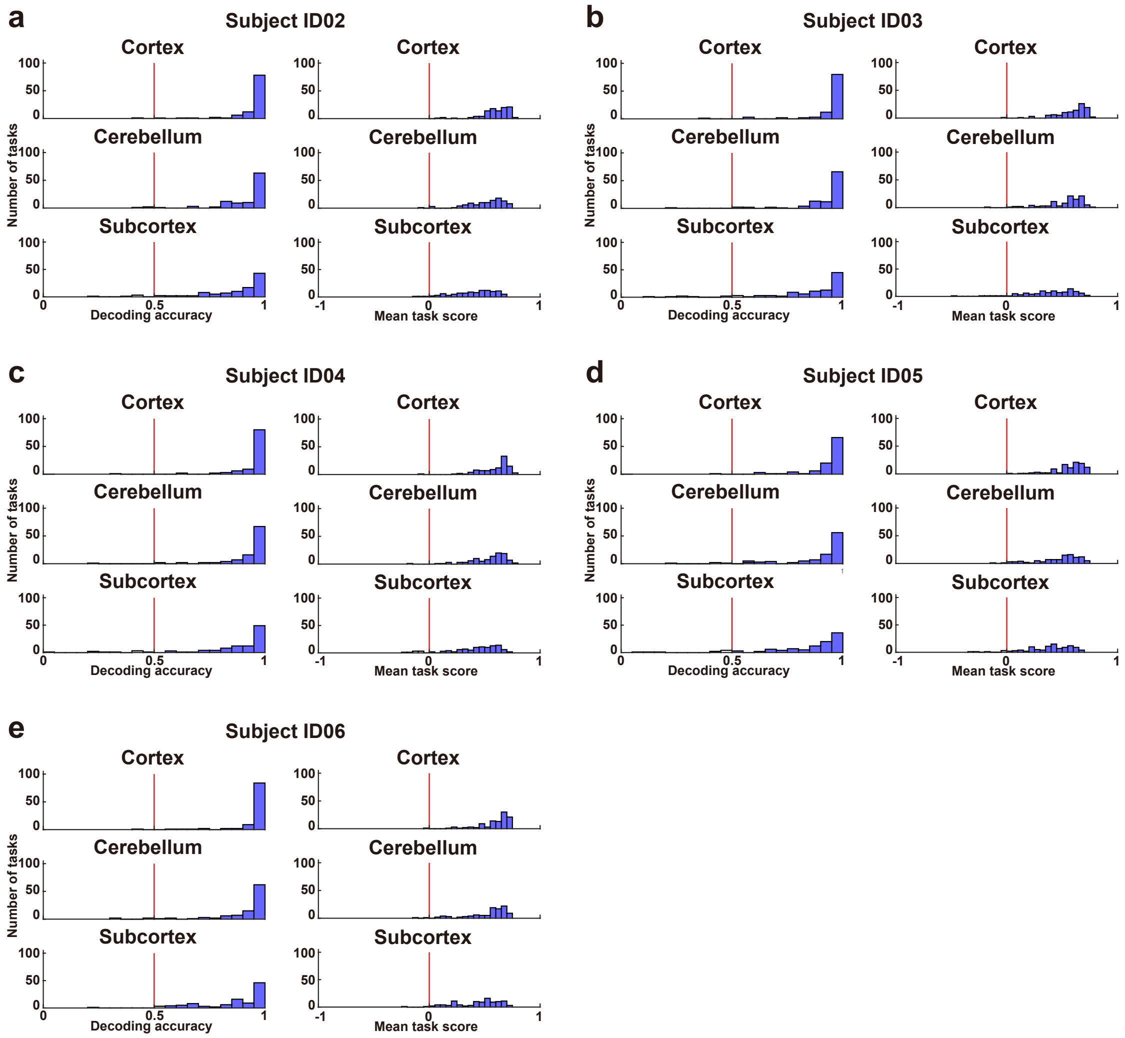

Supplementary Figure 7. Histogram of task decoding accuracies for subject ID02–ID06, using a binary classification (left) and average task score (right). The red line indicates chance-level accuracy (left, 0.5; right, 0). Filled bars indicate tasks that were decoded with significant accuracy ($P < 0.05$, FDR-corrected).

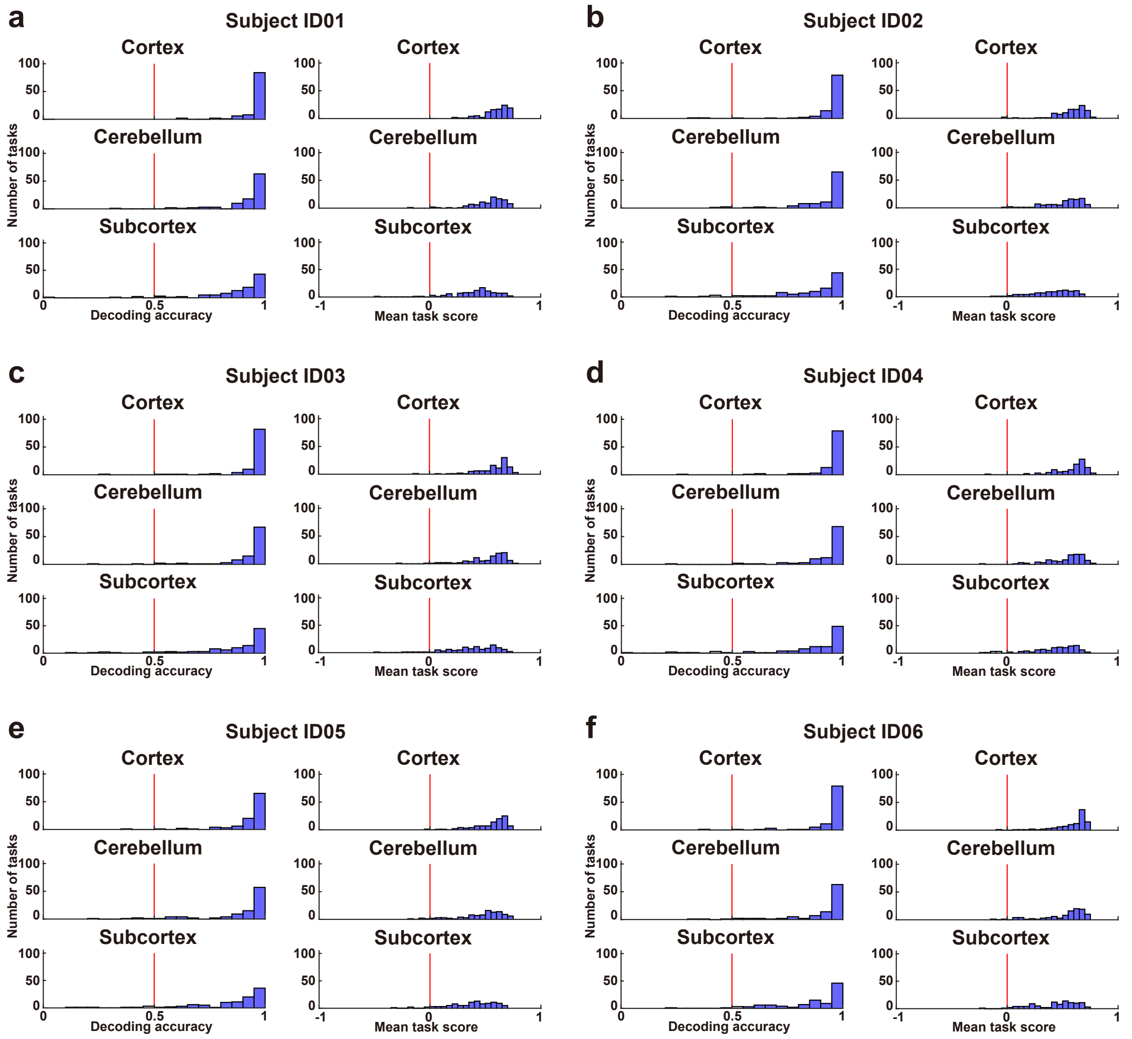

Supplementary Figure 8. Histogram of task decoding accuracies for subjects ID01–ID06, after excluding sensorimotor voxels, using a binary classification (left) and average task score (right). The red line indicates chance-level accuracy (left, 0.5; right, 0). Filled bars indicate tasks that were decoded with significant accuracy (*P* < 0.05, FDR-corrected).

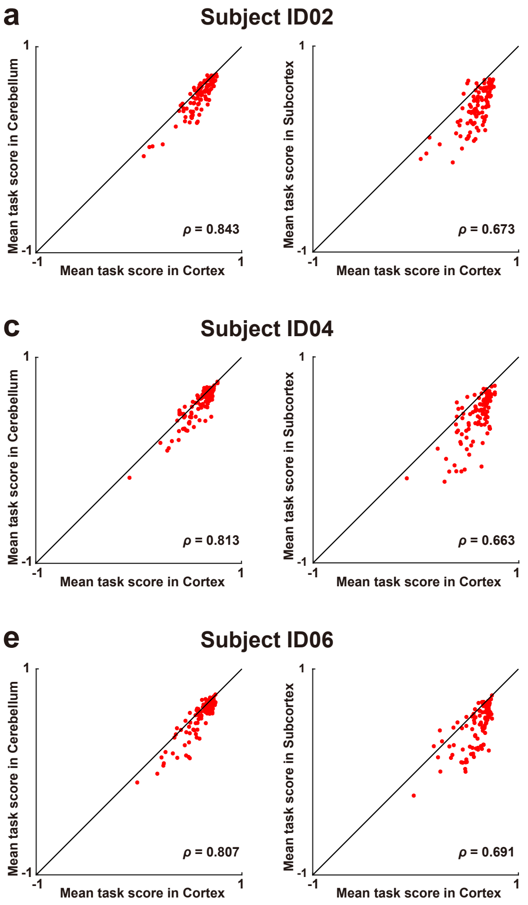

Supplementary Figure 9. Scatter plot of the decoded task score for 103 tasks by the (left) cortex and cerebellum models and (right) cortex and subcortex models. Shown for subject ID02–ID06.

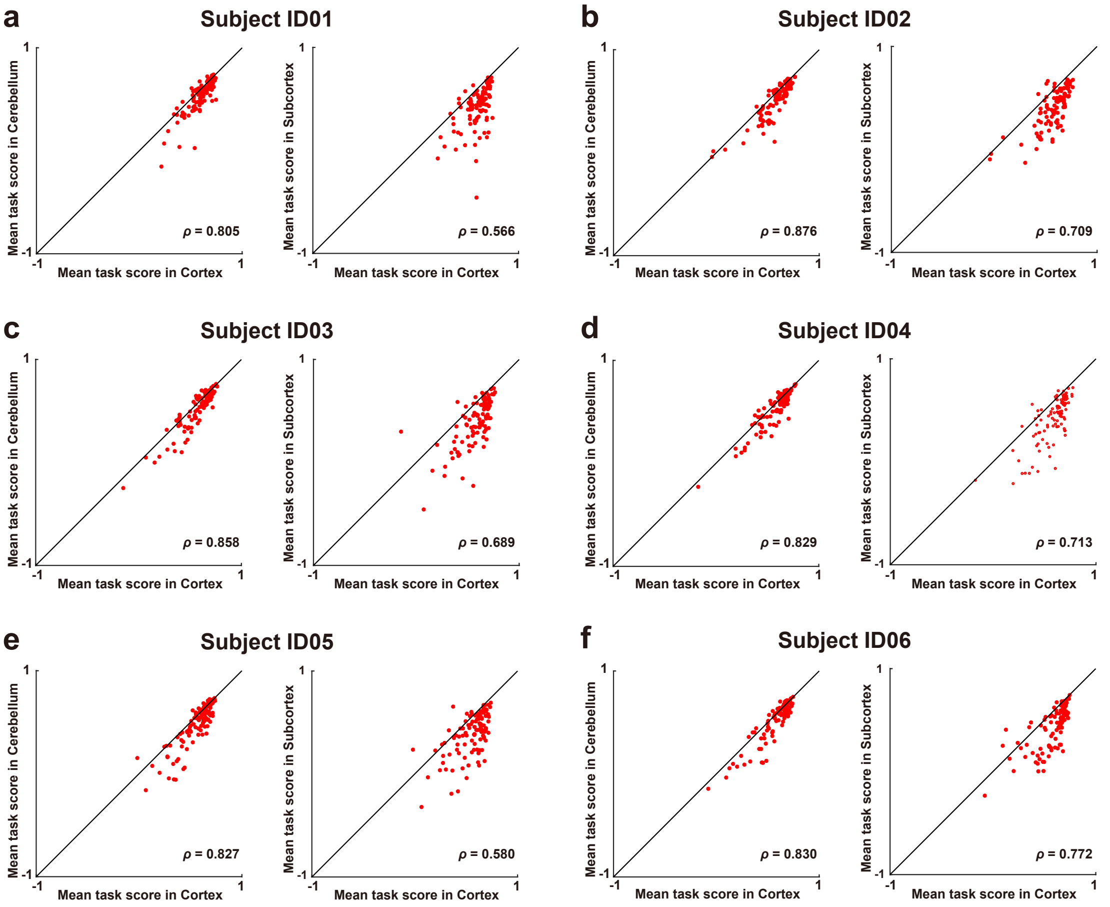

Supplementary Figure 10. Scatter plot of the decoded task score for 103 tasks by the (left) cortex and cerebellum models and (right) cortex and subcortex models after excluding sensorimotor voxels. Shown for subject ID01–ID06.

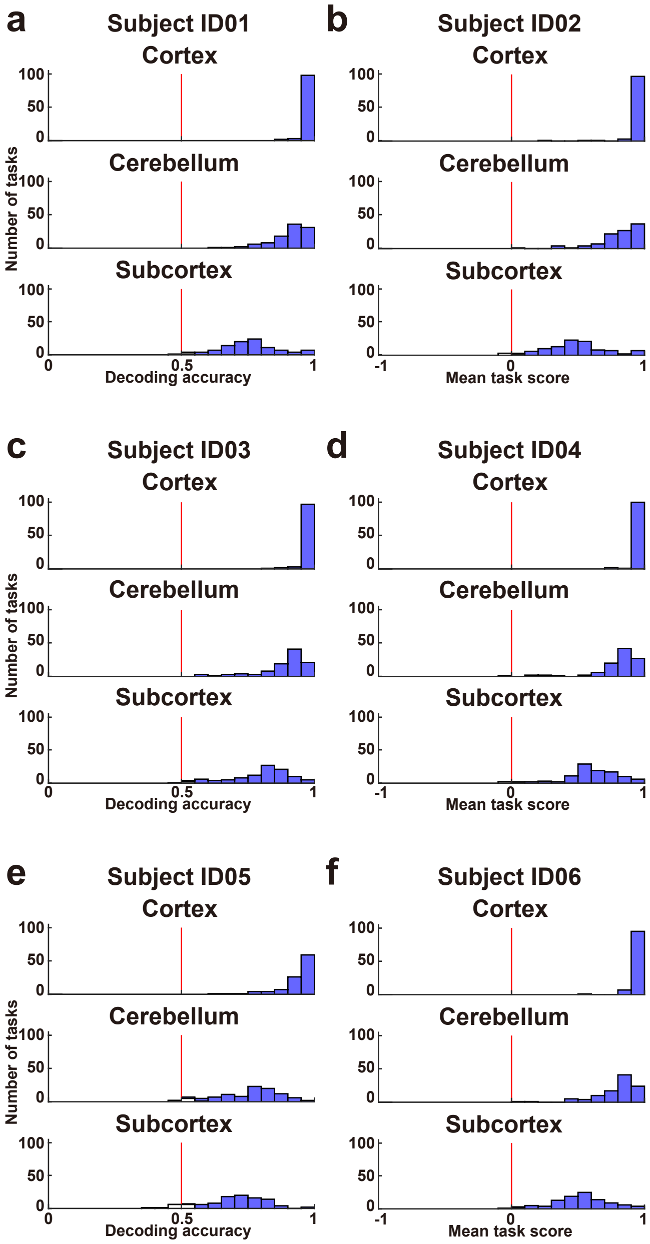

Supplementary Figure 11. Histogram of task decoding accuracies using a support vector machine for subjects ID01–ID06. The red line indicates chance-level accuracy (left, 0.5; right, 0). Filled bars indicate tasks that were decoded with significant accuracy (one-sided sign test, $P < 0.05$, FDR-corrected).

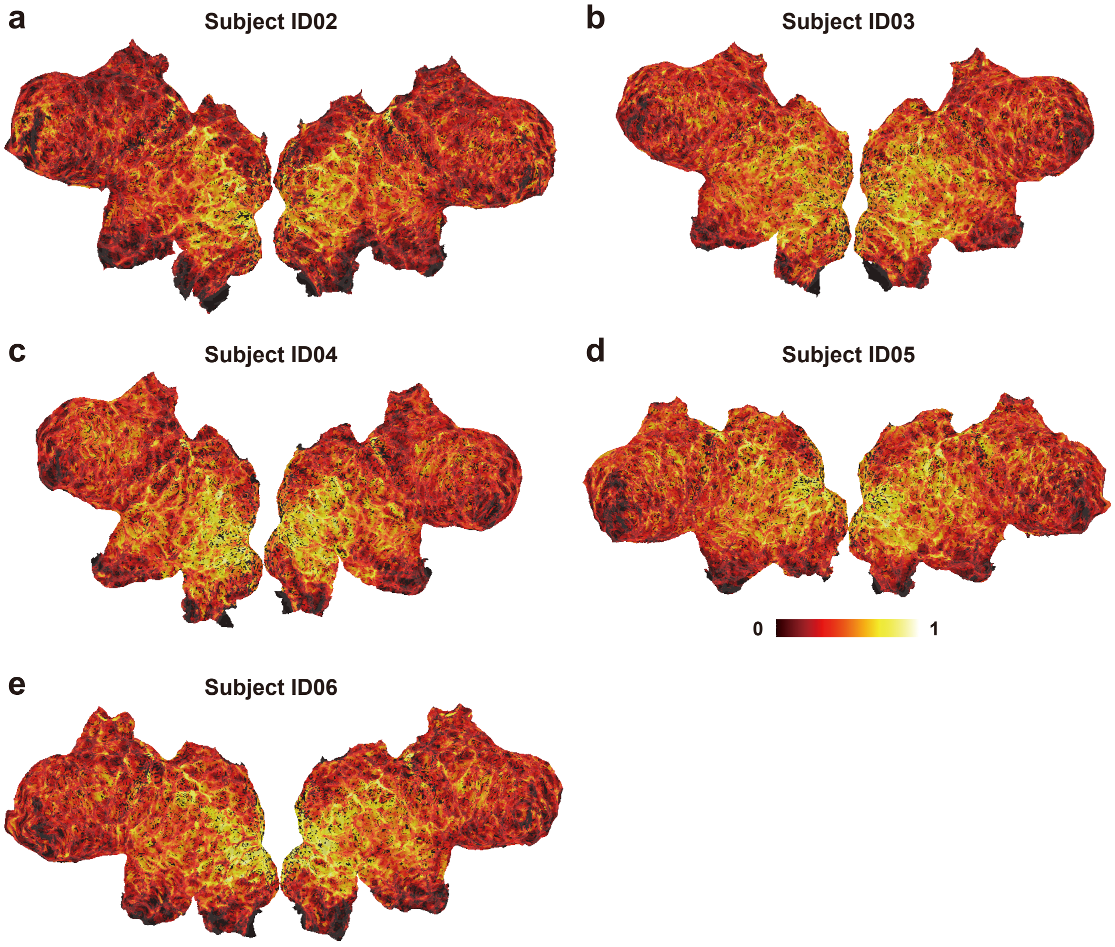

Supplementary Figure 12. Cortical map of model prediction accuracy using cerebellum encoding model, mapped on the flattened cortical sheets of subjects ID02–ID06 ($P < 0.05$, FDR-corrected).

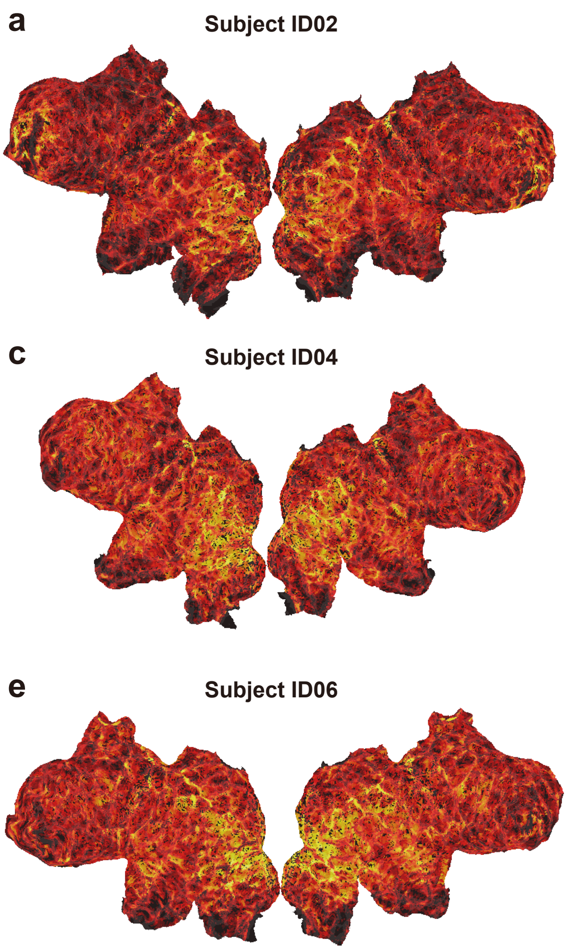

Supplementary Figure 13. Cortical map of model prediction accuracy using the subcortex encoding model, mapped on the flattened cortical sheets of subjects ID02–ID06 ($P < 0.05$, FDR-corrected).

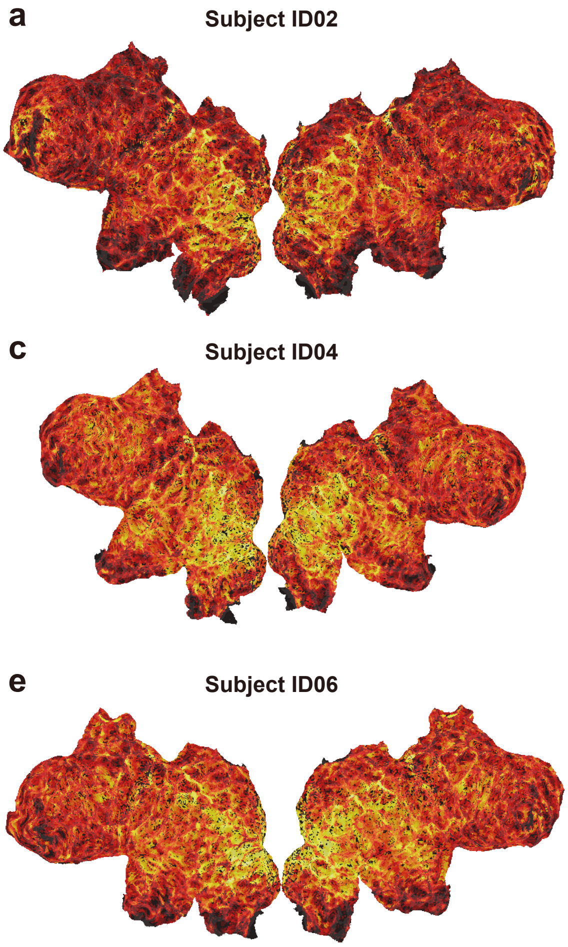

Supplementary Figure 14. Cortical map of model prediction accuracy using the cerebellum + subcortex encoding model, mapped on the flattened cortical sheets of subjects ID02–ID06 ($P <$ 0.05, FDR-corrected).

Supplementary Table 1. Ratios of the removed sensorimotor voxels in representative regions

Regions	Thresholds		
	0.3	0.2	0.1
Whole cortex	18.2% ± 5.0%	35.3% ± 9.1%	63.4% ± 10.1%
Whole cerebellum	1.8% ± 0.8%	7.0% ± 2.7%	33.3% ± 9.1%
Whole subcortex	0.1% ± 0.1%	0.5% ± 0.2%	8.8% ± 3.6%
Left IFG	8.2% ± 6.1%	26.6% ± 16.5%	67.0% ± 18.2%
Right IFG	13.0% + 10.2%	34.8% + 17.4%	73.4% + 13.9%
Left IPL	23.7% ± 11.3%	52.3% ± 18.0%	84.5% ± 9.0%
Right IPL	25.7% + 10.7%	54.7% + 13.2%	86.0% + 5.6%
Left HG	54.7% ± 17.2%	74.0% ± 12.6%	91.3% ± 6.7%
Right HG	56.7% + 17.7%	75.7% + 12.8%	93.9% + 6.1%
Left OP	57.7% ± 5.9%	73.0% ± 6.2%	86.3% ± 7.6%
Right OP	59.3% + 9.1%	72.8% + 8.0%	86.5% + 4.8%

IFG, inferior frontal gyrus; IPL, inferior parietal lobule; HG, Heschl's gyrus; OP, occipital pole.

Supplementary Note 1

Task similarities after excluding sensorimotor voxels with other thresholds

In all thresholds, we found a significant correlation between task similarities of the cortex and cerebellum (threshold = 0.2, ρ = 0.820; threshold = 0.1, ρ = 0.785), as well as the cortex and subcortex (threshold = 0.2, ρ = 0.669; threshold = 0.1, ρ = 0.662).

Decoding after excluding sensorimotor voxels with other thresholds

With threshold = 0.2, most tasks were significantly decoded using the cortical voxels (threshold = 0.2; mean ± SD, 94.6% ± 0.9%; 98.9% ± 0.7% of the tasks were significant; threshold = 0.1; mean ± SD, 93.1% ± 1.2%; 97.6% ± 1.2% of the tasks were significant), cerebellar voxels (threshold = 0.2; mean ± SD, 90.9% ± 1.5%; 96.0% ± 3.0% of the tasks were significant; threshold = 0.1; mean ± SD, 88.6% ± 1.4%; 93.5% ± 3.0% of the tasks were significant), and subcortical voxels (threshold = 0.2; mean ± SD, 85.4% ± 1.2%; 91.9% ± 1.6% of the tasks were significant; threshold = 0.1; mean ± SD, 84.6% ± 1.4%; 90.0% ± 1.7% of the tasks were significant). Positive correlations of decoding performances were again found between the cortex and cerebellum (ρ = 0.839 ± 0.032) and between the cortex and subcortex (ρ = 0.695 ± 0.089).

With threshold = 0.1, most tasks were significantly decoded using the cortical voxels (mean ± SD, 93.1% ± 1.2%; 97.6% ± 1.2% of the tasks were significant), cerebellar voxels (mean ± SD, 88.6% ± 1.4%; 93.5% ± 3.0% of the tasks were significant), and subcortical voxels (threshold = 0.1; mean ± SD, 84.6% ± 1.4%; 90.0% ± 1.7% of the tasks were significant). Positive correlations of decoding performances were again found between the cortex and cerebellum (ρ = 0.825 ± 0.051) and between the cortex and subcortex (ρ = 0.711 ± 0.093).

Supplementary Table 2. Top cognitive factors related to each principal component of the cerebellum

		Top and bottom cognitive factors in the Neurosynth database
PC1	Top	“self referential,” “belief,” “word form,” “disgust,” “visual word,” “mind,” “self,” “theory mind,” “face,” “anger”
	Bottom	“execution,” “working memory,” “verbal working,” “motor,” “imagery,” “memory,” “rehearsal,” “motor imagery,” “visual motion,” “muscle”
PC2	Top	“rehearsal,” “face,” “phonological,” “verbal,” “working memory,” “visual,” “verbal working,” “execution,” “reading,” “task”
	Bottom	“autobiographical,” “autobiographical memory,” “episodic,” “mentalizing,” “mind tom,” “self referential,” “theory mind,” “aging,” “nervous,” “tom”
PC3	Top	“autobiographical,” “sentence,” “content,” “semantic,” “mind,” “reading,” “comprehension,” “empathy,” “default,” “theory mind”
	Bottom	“motor,” “finger,” “movement,” “sensorimotor,” “hand,” “finger movements,” “index finger,” “finger tapping,” “tapping,” “muscle”
PC4	Top	“finger,” “object,” “index finger,” “sensorimotor,” “motor,” “finger movements,” “hand,” “somatosensory,” “movement,” “finger tapping”
	Bottom	“rehearsal,” “reading,” “working memory,” “verbal working,” “verbal,” “sentence,” “linguistic,” “wm task,” “phonological,” “memory”
PC5	Top	“cognitive task,” “phonological,” “semantic,” “calculation,” “impulsivity,” “verbal,” “face,” “navigation,” “executive,” “modality”
	Bottom	“finger,” “motor,” “movement,” “sensorimotor,” “hand,” “tapping,” “finger tapping,” “somatosensory,” “index finger,” “execution”

The top and bottom 10 cognitive factors in the Neurosynth database for PC1–PC5, based on the correlation coefficients between each PC score map and the 715 registered reverse inference maps.

Supplementary Table 3. Top cognitive factors related to each principal component of the subcortex

		Top cognitive factors in the Neurosynth database
PC1	Top	“face,” “encoding,” “emotional,” “happy,” “neutral,” “emotion,” “valence,” “angry,” “episodic memory,” “episodic”
	Bottom	“finger,” “finger tapping,” “tapping,” “sensorimotor,” “execution,” “finger movements,” “motor imagery,” “monetary,” “pain,” “movement”
PC2	Top	“encoding,” “episodic,” “memory,” “autobiographical,” “episodic memory,” “retrieval,” “alzheimer,” “alzheimer disease,” “autobiographical memory,” “subsequent memory”
	Bottom	“pain,” “somatosensory,” “secondary somatosensory,” “noxious,” “painful,” “chronic pain,” “heat,” “finger tapping,” “finger,” “tapping”
PC3	Top	“muscle,” “nervous,” “sensorimotor,” “finger,” “motor performance,” “motor imagery,” “movement,” “motor task,” “pd,” “foot”
	Bottom	“pain,” “emotional,” “painful,” “task,” “face,” “aversive,” “threat,” “affective,” “emotion,” “happy”
PC4	Top	“preparation,” “vocal,” “preparatory,” “pseudowords,” “motor imagery,” “execution,” “parkinson,” “motor,” “finger movements,” “monetary”
	Bottom	“pain,” “somatosensory,” “secondary somatosensory,” “painful,” “positive negative,” “sexual,” “response inhibition,” “intensity,” “positive,” “heat”
PC5	Top	“retrieval,” “episodic,” “memory,” “episodic memory,” “encoding,” “autobiographical,” “retrieved,” “autobiographical memory,” “alzheimer,” “navigation”
	Bottom	“finger,” “movement,” “sensorimotor,” “motor,” “finger tapping,” “execution,” “finger movements,” “tapping,” “hand,” “motor imagery”

The top and bottom 10 cognitive factors in the Neurosynth database for PC1–PC5, based on the correlation coefficients between each PC score map and the 715 registered reverse inference maps.

Supplementary Table 4. Mean decoding accuracy with the one-vs-one method

	ID01	ID02	ID03	ID04	ID05	ID06
	Original					
Cortex	0.965	0.951	0.952	0.953	0.937	0.955
Cerebellum	0.929	0.917	0.926	0.932	0.891	0.911
Subcortex	0.873	0.861	0.849	0.858	0.834	0.860
	After excluding sensorimotor voxels (Thresholds = 0.3)					
Cortex	0.963	0.949	0.951	0.952	0.936	0.948
Cerebellum	0.925	0.920	0.923	0.926	0.888	0.907
Subcortex	0.872	0.862	0.849	0.858	0.838	0.858

Supplementary Table 5. Percentage of significant tasks with the one-vs-one method

	ID01	ID02	ID03	ID04	ID05	ID06
	Original					
Cortex	100.0%	99.0%	100.0%	100.0%	100.0%	99.0%
Cerebellum	100.0%	97.1%	97.1%	98.1%	94.2%	96.1%
Subcortex	94.2%	93.2%	90.3%	91.3%	91.3%	96.1%
	After excluding sensorimotor voxels (Thresholds = 0.3)					
Cortex	100.0%	98.1%	99.0%	100.0%	99.0%	99.0%
Cerebellum	100.0%	97.1%	96.1%	98.1%	94.2%	95.1%
Subcortex	94.2%	93.2%	90.3%	91.3%	90.3%	95.1%

Supplementary Table 6. Mean decoding accuracy with the task score method

	ID01	ID02	ID03	ID04	ID05	ID06
	Original					
Cortex	0.615	0.597	0.591	0.594	0.560	0.595
Cerebellum	0.540	0.517	0.526	0.538	0.467	0.529
Subcortex	0.413	0.399	0.388	0.415	0.379	0.432
	After excluding sensorimotor voxels (Thresholds = 0.3)					
Cortex	0.601	0.580	0.577	0.583	0.551	0.584
Cerebellum	0.532	0.509	0.517	0.528	0.463	0.522
Subcortex	0.412	0.399	0.387	0.415	0.380	0.431

Supplementary Table 7. Percentage of significant tasks with the task score method

	ID01	ID02	ID03	ID04	ID05	ID06
	Original					
Cortex	100.0%	100.0%	99.0%	99.0%	100.0%	99.0%
Cerebellum	99.0%	99.0%	99.0%	99.0%	98.1%	98.1%
Subcortex	97.1%	97.1%	94.2%	92.2%	94.2%	98.1%
	After excluding sensorimotor voxels (Thresholds = 0.3)					
Cortex	100.0%	98.1%	99.0%	99.0%	99.0%	99.0%
Cerebellum	99.0%	99.0%	98.1%	99.0%	96.1%	98.1%
Subcortex	97.1%	97.1%	94.2%	92.2%	95.1%	98.1%

Supplementary Table 8. Mean decoding accuracy using the support vector machine

	ID01	ID02	ID03	ID04	ID05	ID06
	Original					
Cortex	98.7%	97.6%	98.1%	98.7%	93.3%	98.3%
Cerebellum	90.6%	90.5%	88.6%	90.1%	75.3%	89.7%
Subcortex	75.0%	73.7%	79.2%	80.1%	69.9%	75.8%

Supplementary Table 9. Percentage of significant tasks using the support vector machine

	ID01	ID02	ID03	ID04	ID05	ID06
	Original					
Cortex	100.0%	100.0%	100.0%	100.0%	100.0%	100.0%
Cerebellum	100.0%	100.0%	100.0%	100.0%	92.2%	100.0%
Subcortex	96.1%	95.1%	97.1%	97.1%	86.4%	98.1%

Supplementary Note 2

Description of each task

1. PressRight

Subjects pressed the buttons (with their right hand) as many times as possible. Duration: 8 s.

2. PressLeft

Subjects pressed the buttons (with their left hand) as many times as possible. Duration: 8 s.

3. PressLR

Subjects pressed the buttons (with their right or left hand) as many times as possible. Duration: 8 s.

4. RestOpen

Subjects did not perform any task and kept their eyes open. Duration: 10 s.

5. RestClose

Subjects did not perform any task and kept their eyes closed. Duration: 10 s.

6. EyeBlink

Subjects blinked their eyes as many times as possible. Duration: 8 s.

7. RateTired

Subjects rated how tired they were by pressing one of the four buttons. Duration: 6 s.

8. RateConfidence

Subjects rated how confident they were about their accuracy on the previous task by pressing one of the four buttons. Duration: 6 s.

9. RateSleepy

Subjects rated how sleepy they were by pressing one of the four buttons. Duration: 6 s.

10. ImagineFuture

Subjects imagined their future situation (e.g., "Imagine your next weekend"). Duration: 8 s.

11. ImagineIf

Subjects imagined they were some other living thing. Duration: 8 s.

12. ImagineMove

Subjects imagined their body moving. Duration: 8 s.

13. ImaginePlace

Subjects imagined a certain place. Duration: 8 s.

14. RecallPast

Subjects recalled a past event. Duration: 8 s.

15. RecallKnowledge

Subjects recalled as many names as possible that have a given property (e.g., recall as many Japanese river names as possible). Duration: 10 s.

16. RecallFace

Subjects recalled the face of somebody. Duration: 8 s.

17. LetterFluency

Subjects recalled as many words as possible starting with a given letter. Duration: 10 s.

18. CategoryFluency

Subjects recalled as many words as possible belonging to a given word category. Duration: 10 s.

19. Clock

Subjects looked at a photo of a clock and judged whether the indicated time matched the time displayed above the photo. Duration: 6 s.

20. AnimalPhoto

Subjects looked at a photo of an animal and judged whether its name matched the name displayed above the photo. Duration: 6 s.

21. AnimalVoice

Subjects listened to the voice of an animal and judged whether its name matched the name shown on the screen. Duration: 6 s.

22. Money

Subjects looked at a photo of money and judged whether the indicated amount matched the amount displayed above the photo. Duration: 8 s.

23. TrafficSign

Subjects looked at a photo of a traffic sign and judged whether its meaning matched the meaning indicated above the photo. Duration: 6 s.

24. EmotionFace

Subjects looked at a photo of a face with a specific emotion and judged whether the emotion matched the emotion indicated above the photo. Duration: 6 s.

25. EmotionVoice

Subjects listened to a voice with a specific emotion and judged whether the emotion matched the emotion indicated above the photo. Duration: 6 s.

26. Flag

Subjects looked at a photo of a national flag and judged whether the country matched the country indicated above the photo. Duration: 6 s.

27. MapSymbol

Subjects looked at a photo of a map symbol and judged whether its meaning matched the meaning indicated above the photo. Duration: 6 s.

28. CalcEasy

Subjects solved an easy arithmetic problem using single digits. Duration: 8 s.

29. CalcHard

Subjects solved a difficult arithmetic problem using two-digit numbers. Duration: 10 s.

30. DailyPhoto

Subjects looked at a photo of a tool used daily and judged whether its name matched the name displayed above the photo. Duration: 6 s.

31. DailySound

Subjects listened to the sound of tool used daily and judged whether its name matched the name displayed above the photo. Duration: 6 s.

32. CountDot

Subjects counted the number of presented dots. Duration: 8 s.

33. CountTone

Subjects counted the number of presented tones. Duration: 8 s.

34. CountryMap

Subjects looked at a photo of a country map and judged whether its name (nation) matched the name displayed above the photo. Duration: 6 s.

35. StateMap

Subjects looked at a photo of a state (prefecture) map and judged whether its name matched the name displayed above the photo. Duration: 6 s.

36. RateSexyPicF

Subjects looked at a photo of a female and rated how sexy they thought she was. Duration: 6 s.

37. RateSexyPicM

Subjects looked at a photo of a male and rated how sexy they thought he was. Duration: 6 s.

38. RateSexyMovM

Subjects viewed a movie of a male and rated how sexy they thought he was. Duration: 10 s.

39. RateSexyMovF

Subjects viewed a movie of a female and rated how sexy they thought she was. Duration: 10 s.

40. RateBeautyPic

Subjects looked at a photo and rated how beautiful they thought it was. Duration: 6 s.

41. RateBeautySound

Subjects listened to a piece of music and rated how beautiful they thought it was. Duration: 10 s.

42. RateBeautyMov

Subjects viewed a movie and rated how beautiful they thought it was. Duration: 10 s.

43. RateDisgustPic

Subjects looked at a photo and rated how disgusting they thought it was. Duration: 6 s.

44. RateDisgustSound

Subjects listened to a sound and rated how disgusting they thought it was. Duration: 6 s.

45. RateDisgustMov

Subjects viewed a movie and rated how disgusting they thought it was. Duration: 10 s.

46. RateHappyPic

Subjects looked at a photo and rated how happy the situation seemed to be. Duration: 6 s.

47. RateHappyMov

Subjects viewed a movie and rated how happy the situation seemed to be. Duration: 10 s.

48. RateDeliciousPic

Subjects saw a photo of food and rated how delicious it looked. Duration: 6 s.

49. RateDeliciousMov

Subjects viewed a movie of food and rated how delicious it looked. Duration: 10 s.

50. RatePainfulPic

Subjects looked at a photo and rated how painful the situation seemed to be. Duration: 6 s.

51. RatePainfulMov

Subjects viewed a movie and rated how painful the situation seemed to be. Duration: 10 s.

52. RateNoisy

Subjects listened to a sound and rated how noisy they thought it was. Duration: 8 s.

53. RatePoem

Subjects read a poem and rated how good they thought it was. Duration: 12 s.

54. WordMeaning

Subjects judged whether the meaning of a presented word matched the sentence displayed above the word. Duration: 6 s.

55. EyeMoveEasy

Subjects looked at a small circle moving around in 1 Hz. Duration: 8 s.

56. EyeMoveHard

Subjects looked at a small circle moving around at 2 Hz. Duration: 8 s.

57. WorldName

Subjects looked at the photo of a foreign celebrity and judged whether their name matched the name displayed above the photo. Duration: 6 s.

58. DomesticName

Subjects looked at the photo of a local celebrity and judged whether their name matched the name displayed above the photo. Duration: 6 s.

59. SoundPlace

Subjects listened to an environmental sound and judged whether it matched the location on the

screen. Duration: 6 s.

60. WorldPlace

Subjects looked at a photo of a place in some foreign country and judged whether it matched the site displayed above the photo. Duration: 6 s.

61. DomesticPlace

Subjects looked at a photo of a place in their home country and judged whether it matched the site displayed above the photo. Duration: 6 s.

62. MusicCategory

Subjects judged whether the genre of a piece of music matched the name displayed on the screen. Duration: 10 s.

63. DetectTargetPic

Subjects judged whether a target item was shown in a photo. Duration: 8 s.

64. DetectTargetMov

Subjects judged whether a target item was shown in a movie clip. Duration: 10 s.

65. Metaphor

Subjects read a metaphorical text and judged whether the writer's intention matched the meaning indicated above the text. Duration: 8 s.

66. Sarcasm

Subjects read a sarcastic text and judged whether the writer's intention matched the meaning indicated above the text. Duration: 8 s.

67. TimeMov

Subjects judged whether the duration of a presented movie matched the duration indicated on the screen. Duration: 8 s.

68. TimeSound

Subjects judged whether the duration of a presented sound matched the duration indicated on the screen. Duration: 8 s.

69. ComparePeople

Subjects looked at two photos of people and judged whether or not the two were the same person. Duration: 6 s.

70. DetectDifference

Subjects looked at two pictures and judged whether or not they were exactly the same. Duration: 8 s.

71. Harmony

Subjects listened to a sequence of chords and judged whether the chord progression was consonant or dissonant. Duration: 6 s.

72. DecideFood

Subjects looked at four photos of different foods and judged which looked the most delicious. Duration: 8 s.

73. DecidePeople

Subjects looked at four photos of different people and judged who looked the most reliable. Duration: 8 s.

74. DecidePresent

Subjects chose one among four items they wanted to receive as a present. Duration: 8 s.

75. DecideShopping

Subjects chose one among four items they would buy during shopping. Duration: 8 s.

76. LanguageSound

Subjects listened to a sound and judged whether the language matched the language indicated on the screen. Duration: 6 s.

77. DetectColor

Subject judged whether the color of a word matched the color displayed above the word. Duration: 6 s.

78. SoundLeft

Subjects judged whether a sound was presented from their left side. Duration: 8 s.

79. SoundRight

Subjects judged whether a sound was presented from their right side. Duration: 8 s.

80. RelationLogic

Subjects read a syllogism based on spatial relationships and indicated whether the conclusion was valid or not. Duration: 12 s.

81. PropLogic

Subjects read a syllogism based on prepositional logical relationships and indicated whether the conclusion was valid or not. Duration: 12 s.

82. MoralPersonal

Subjects read a text and judged whether the described activity (which included harming somebody) was ethically permissible or not. Duration: 12 s.

83. MoralImpersonal

Subjects read a text and judged whether the described activity (which did not include harming somebody) was ethically permissible or not. Duration: 12 s.

84. Recipe

Subjects judged whether a given recipe matched the actual recipe of a given dish. Duration: 8 s.

85. TimeValue

Subjects selected one of two money rewards which would be offered to them at different points in the future. Duration: 8 s.

86. PressOrdEasy

Subjects pressed buttons based on a series of numbers presented at 1 Hz. Duration: 8 s.

87. PressOrdHard

Subjects pressed buttons based on a series of numbers presented at 2 Hz. Duration: 8 s.

88. Rhythm

Subjects listened to a series of sound pulses and judged whether its rhythm was constant or not. Duration: 6 s.

89. RecallTaskEasy

Subjects judged whether the two earlier tasks matched the task described on the screen. Duration: 6 s.

90. RecallTaskHard

Subjects judged whether the three earlier tasks matched the task described on the screen. Duration: 6 s.

91. MemoryDigit

Subjects memorized a series of digits. Duration: 6 s.

92. MatchDigit

Subjects judged whether a presented series of digits matched the one presented before (corresponding to the digits memorized in the MemoryDigit task). Duration: 6 s.

93. MemoryLetter

Subjects memorized a series of letters. Duration: 6 s.

94. MatchLetter

Subjects judged whether a presented series of letters matched the one presented before (corresponding to the letters memorized in the MemoryLetter task). Duration: 6 s.

95. MemoryNameEasy

Subjects memorized three names associated with three photos of different animal species. Duration: 6 s.

96. MatchNameEasy

Subjects judged whether two presented photos matched the names displayed on the screen (corresponding to the names memorized in the MemoryNameEasy task). Duration: 8 s.

97. MemoryNameHard

Subjects memorized three names associated with three photos of the same animal species. Duration: 6 s.

98. MatchNameHard

Subjects judged whether two presented photos matched the names displayed on the screen (corresponding to the names memorized in the MemoryNameHard task). Duration: 8 s.

99. ForeignRead

Subjects read an English sentence (i.e., a foreign language for the subjects). Duration: 12 s.

100. ForeignReadQ

Subjects answered a question about the English sentence they read just before. Duration: 6 s.

101. ForeignListen

Subjects listened to an English sentence. Duration: 10 s.

102. ForeignListenQ

Subjects answered a question about the English sentence they listened to just before. Duration: 6 s.

103. MirrorImage

Subjects judged whether a photo was symmetrical or not. Duration: 6 s.

Supplementary References

1. King, M., Hernandez-Castillo, C. R., Poldrack, R. A., Ivry, R. B. & Diedrichsen, J. Functional boundaries in the human cerebellum revealed by a multi-domain task battery. *Nat. Neurosci.* **22**, 1371–1378 (2019).
