# Supplementary material for: Representations and decodability of diverse cognitive functions are preserved across the human cortex, cerebellum, and subcortex
Source: Commun Biol. 2022 Nov 14;5:1245. doi: 10.1038/s42003-022-04221-y (PMC9663596; doi:10.1038/s42003-022-04221-y)
Supplement: Supplementary file 3 — Description of Additional Supplementary Files [file 42003_2022_4221_MOESM3_ESM.pdf]

Description of Additional Supplementary Files

File name: Supplementary Data 1

Description: The source data behind the Fig. 2d-f in the paper

File name: Supplementary Data 2

Description: The source data behind the Fig. 3b in the paper

File name: Supplementary Data 3

Description: The source data behind the Fig.4b in the paper

File name: Supplementary Data 4

Description: The source data behind the Fig. 5b-e in the paper

File name: Supplementary Data 5

Description: The source data behind the Fig. 5f in the paper
