# Supplementary material for: Representations and decodability of diverse cognitive functions are preserved across the human cortex, cerebellum, and subcortex
Source: Commun Biol. 2022 Nov 14;5:1245. doi: 10.1038/s42003-022-04221-y (PMC9663596; doi:10.1038/s42003-022-04221-y)
Supplement: Supplementary file 9 — Reporting Summary [file 42003_2022_4221_MOESM9_ESM.pdf]

# nature portfolio

Corresponding author(s): Tomoya Nakai
Last updated by author(s): Oct 27, 2022

# Reporting Summary

Nature Portfolio wishes to improve the reproducibility of the work that we publish. This form provides structure for consistency and transparency in reporting. For further information on Nature Portfolio policies, see our Editorial Policies and the Editorial Policy Checklist.

## Statistics

For all statistical analyses, confirm that the following items are present in the figure legend, table legend, main text, or Methods section.

| n/a | Confirmed | |
|---|---|---|
| ☐ | ☒ | The exact sample size ($n$) for each experimental group/condition, given as a discrete number and unit of measurement |
| ☐ | ☒ | A statement on whether measurements were taken from distinct samples or whether the same sample was measured repeatedly |
| ☐ | ☒ | The statistical test(s) used AND whether they are one- or two-sided<br>*Only common tests should be described solely by name; describe more complex techniques in the Methods section.* |
| ☐ | ☒ | A description of all covariates tested |
| ☐ | ☒ | A description of any assumptions or corrections, such as tests of normality and adjustment for multiple comparisons |
| ☐ | ☒ | A full description of the statistical parameters including central tendency (e.g. means) or other basic estimates (e.g. regression coefficient) AND variation (e.g. standard deviation) or associated estimates of uncertainty (e.g. confidence intervals) |
| ☐ | ☒ | For null hypothesis testing, the test statistic (e.g. $F$, $t$, $r$) with confidence intervals, effect sizes, degrees of freedom and $P$ value noted<br>*Give P values as exact values whenever suitable.* |
| ☒ | ☐ | For Bayesian analysis, information on the choice of priors and Markov chain Monte Carlo settings |
| ☒ | ☐ | For hierarchical and complex designs, identification of the appropriate level for tests and full reporting of outcomes |
| ☐ | ☒ | Estimates of effect sizes (e.g. Cohen's $d$, Pearson's $r$), indicating how they were calculated |

*Our web collection on statistics for biologists contains articles on many of the points above.*

## Software and code

Policy information about availability of computer code

| | |
|---|---|
| Data collection | Siemens MAGNETOM TrioTim (Siemens, syngo MR B17) and Presentation (Neurobehavioral Systems, ver. 18.0) |
| Data analysis | We used MATLAB (MathWorks Inc., 2016b) to estimate model weights of a multivariate regression model (Nishimoto et al., 2011 Current Biology; Huth et al., 2012 Neuron).<br>Visualization of the model weights on the cortical map was conducted using pycortex (https://github.com/gallantlab/pycortex).<br>Representational similarity analysis and principal component analysis were performed using the standard MATLAB functions. |

For manuscripts utilizing custom algorithms or software that are central to the research but not yet described in published literature, software must be made available to editors and reviewers. We strongly encourage code deposition in a community repository (e.g. GitHub). See the Nature Portfolio guidelines for submitting code & software for further information.

## Data

Policy information about availability of data

All manuscripts must include a data availability statement. This statement should provide the following information, where applicable:

- Accession codes, unique identifiers, or web links for publicly available datasets
- A description of any restrictions on data availability
- For clinical datasets or third party data, please ensure that the statement adheres to our policy

The raw MRI data are available at the OpenNeuro.org (https://openneuro.org/datasets/ds002306).



March 2021

## Human research participants

Policy information about studies involving human research participants and Sex and Gender in Research.

| | |
|---|---|
| Reporting on sex and gender | Both male (N = 4) and female (N = 2) subjects were included. Sex was determined based on self-reporting. Gender-based analyses were not performed because data for each participant were analyzed individually, and group-level analysis was not the primary objective of this study. |
| Population characteristics | Six healthy participants (aged 22-33 yeas; two females) with normal vision and normal hearing. |
| Recruitment | Participants were recruited from a local participant pool under the following selection criteria: (1) a participant can join at least three fMRI sessions and (2) a participant is healthy and normal vision and normal hearing. |
| Ethics oversight | This experiment was approved by the ethics and safety committee of the National Institute of Information and Communications Technology in Osaka, Japan |

Note that full information on the approval of the study protocol must also be provided in the manuscript.

# Field-specific reporting

Please select the one below that is the best fit for your research. If you are not sure, read the appropriate sections before making your selection.

☒ Life sciences ☐ Behavioural & social sciences ☐ Ecological, evolutionary & environmental sciences

For a reference copy of the document with all sections, see nature.com/documents/nr-reporting-summary-flat.pdf

# Life sciences study design

All studies must disclose on these points even when the disclosure is negative.

| | |
|---|---|
| Sample size | The sample size of the test data was determined to match or exceed our prior attempts (e.g., Nishimoto et al., 2011 Current Biology) |
| Data exclusions | Some data were excluded (and re-measured) when we detected the following technical issues during experiments: the earphone was not properly attached. |
| Replication | Replication was confirmed via high prediction performance of the multivariate model for each and every participant. |
| Randomization | n/a (Participants were not allocated into experimental groups.) |
| Blinding | n/a (Participants were not allocated into experimental groups.) |

# Reporting for specific materials, systems and methods

We require information from authors about some types of materials, experimental systems and methods used in many studies. Here, indicate whether each material, system or method listed is relevant to your study. If you are not sure if a list item applies to your research, read the appropriate section before selecting a response.

### Materials & experimental systems

| n/a | Involved in the study |
|---|---|
| ☒ | ☐ Antibodies |
| ☒ | ☐ Eukaryotic cell lines |
| ☒ | ☐ Palaeontology and archaeology |
| ☒ | ☐ Animals and other organisms |
| ☒ | ☐ Clinical data |
| ☒ | ☐ Dual use research of concern |

### Methods

| n/a | Involved in the study |
|---|---|
| ☒ | ☐ ChIP-seq |
| ☒ | ☐ Flow cytometry |
| ☐ | ☒ MRI-based neuroimaging |

## Magnetic resonance imaging

### Experimental design

| | |
|---|---|
| Design type | Building voxel-wise encoding models using task-evoked brain activity (Nishimoto et al., 2011 Current Biology) |

Design specifications

The main experiment was conducted in three separate fMRI sessions. The total of 18 runs were acquired across the three sessions. Of these, 12 runs were used to train voxel-wise models, and 6 runs were used to test the modeling accuracy. A single run consisted of 556 seconds. Stimuli in the training and test runs were different.

Behavioral performance measures

Eye movements were monitored to confirm participants' arousal level during the experiments, but not used for our analyses.

## Acquisition

Imaging type(s)

functional and structural

Field strength

3T

Sequence & imaging parameters

Functional data: A multiband gradient echo-planar imaging sequence (TR = 2,000 ms, TE = 30 ms, flip angle = 62°; voxel size = 2 × 2 × 2 mm3, matrix size = 96 × 96, 72 axial slices, FOV = 192 × 192 mm2, multiband factor = 3).
Structural data: T1-weighted MPRAGE (TR = 2530 ms, TE = 3.26 ms, flip angle = 9°, voxel size = 1 × 1 × 1 mm3, matrix size = 256 × 256, 256 axial slices, FOV = 256 × 256 mm2).

Area of acquisition

A whole brain scan

Diffusion MRI ☐ Used ☒ Not used

## Preprocessing

Preprocessing software

SPM12 (motion correction) and FreeSurfer 5.3.0 (anatomical registration, cortical surface reconstruction, cortical segmentation, and subcortical segmentation)

Normalization

n/a (data for each participant were analyzed individually)

Normalization template

n/a (data for each participant were analyzed individually)

Noise and artifact removal

Motion correction (6DOF) was performed by aligning all of the EPI data to the first image from the first scan for each subject. For each voxel, responses were normalized by subtracting the mean response across all time points, and trend was removed using a median filter (240-s time window). These processes were performed using in-house MATLAB codes (Cukur et al., 2016 The Journal of Neuroscience). No spatial smoothing procedure was performed.

Volume censoring

We used voxels in the cortex, cerebellum, and subcortex, that were anatomically defined by using FreeSurfer.

## Statistical modeling & inference

Model type and settings

Mass univariate, predictive

Effect(s) tested

Prediction accuracy and decoding accuracy under novel task conditions

Specify type of analysis: ☒ Whole brain ☐ ROI-based ☐ Both

Statistic type for inference
(See Eklund et al. 2016)

voxel-wise

Correction

False-discovery rate (FDR) correction (Benjamini and Hochberg, 1995).

## Models & analysis

| n/a | Involved in the study |
|---|---|
| ☒ | ☐ Functional and/or effective connectivity |
| ☒ | ☐ Graph analysis |
| ☐ | ☒ Multivariate modeling or predictive analysis |

Multivariate modeling and predictive analysis

We built voxel-wise encoding models (Naselaris et al., 2011 NeuroImage; Nishimoto et al., 2011 Current Biology) to explain the BOLD responses using emotion ratings. Model weights were estimated using a L2-regularized linear regression procedure (Huth et al., 2012 Neuron) for training data (3336 samples). The regularization parameter was optimized via 10-fold cross validation using the training data. The prediction accuracy of each voxel model was quantified by a Pearson's correlation coefficients between the measured and the predicted BOLD responses for test data (412 samples).
